# Trisacryl Gelatin Microembolism and Metastases in the Lung after Renal Artery Embolization and Nephrectomy for Renal Cell Carcinoma

**DOI:** 10.1155/2015/916268

**Published:** 2015-05-31

**Authors:** Andres Borja Alvarez, Jack P. Leventhal, Cherise Cortese, Barbara L. McComb, David D. Thiel, Andras Khoor

**Affiliations:** ^1^Division of Pulmonary and Allergy Medicine, Mayo Clinic, Jacksonville, FL 32224, USA; ^2^Department of Laboratory Medicine and Pathology, Mayo Clinic, Jacksonville, FL 32224, USA; ^3^Department of Radiology, Mayo Clinic, Jacksonville, FL 32224, USA; ^4^Department of Urology, Mayo Clinic, Jacksonville, FL 32224, USA

## Abstract

This is the first report, to our knowledge, of widespread, histologically confirmed trisacryl gelatin pulmonary microembolism after renal artery embolization (RAE). In addition, this is the first report of lung involvement by both metastatic renal cell carcinoma (RCC) and an embolic agent used for RAE. The patient was a 63-year-old woman who recently presented with both dyspnea on exertion and productive cough. Her past medical history included clear cell RCC, which was treated with preoperative trisacryl gelatin microsphere RAE and right nephrectomy 9 years earlier. Computed tomography of the chest showed multiple lung nodules, a mass-like density in the left lower lobe, and mediastinal and hilar lymphadenopathy. Wedge resections of the lung showed multiple foci of metastatic RCC and extensive involvement of the muscular pulmonary arteries by trisacryl gelatin microspheres.

## 1. Introduction

Localized renal cell carcinomas (RCCs) are usually treated with total or partial nephrectomy or nephron-sparing surgery. Minimally invasive modalities, including percutaneous radiofrequency ablation and high-intensity ultrasonography, are also available [1]. Renal artery embolization (RAE) can be performed preoperatively to reduce the need for blood transfusion when the tumor is large and highly vascularized [2]. One of the most commonly applied embolic agents is trisacryl gelatin microspheres [3].

## 2. Case Presentation

A 63-year-old woman who had never smoked and previously was preparing to run a 5 km race presented with dyspnea on exertion, chest tightness, palpitations, and productive cough. She denied hemoptysis, weight loss, fever, chills, or night sweats. Nine years earlier, she underwent RAE and right nephrectomy for a 10.5 cm clear cell RCC. Preembolization imaging showed a large strikingly hypervascular right renal mass, with many contributing arteries. There was no evidence of arteriovenous malformation. The embolization procedure with 700–900-micron spherical particles resulted in extensive devascularization of the tumor. The primary tumor had been staged at stage T3a on the basis of invasion of perirenal adipose tissue. No tumor extension into the renal vein or vena cava was detected, but trisacryl gelatin microspheres were noted in renal artery branches.

**Figure 1:**
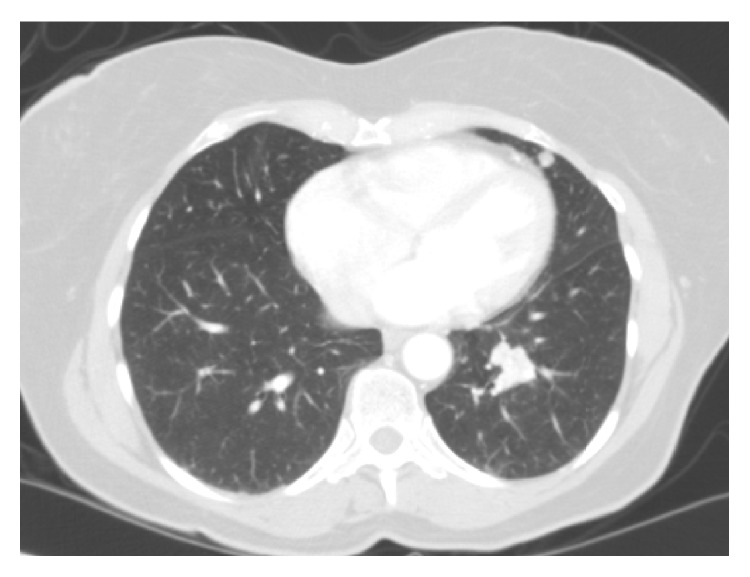
Imaging study. Computed tomography of the chest reveals a 2.0 × 1.6 cm mass and a stable nodule in the left lower lobe.

Vital signs and physical examination were unremarkable. A chest radiograph showed a mass-like density in the left lower lobe with bulky, large mediastinal and left hilar lymphadenopathy and multiple nodules not present on previous imaging. Computed tomography of the chest with contrast medium was notable for a density in the left lower lobe, mediastinal and hilar lymphadenopathy, and multiple nodules (Figure 1). Bronchoscopy showed a partially obstructive lesion in the left mainstem bronchus that bled with suctioning; bronchoalveolar lavage was negative and endoscopic ultrasonographic biopsy of the lymph nodes was nondiagnostic.

**Figure 2:**
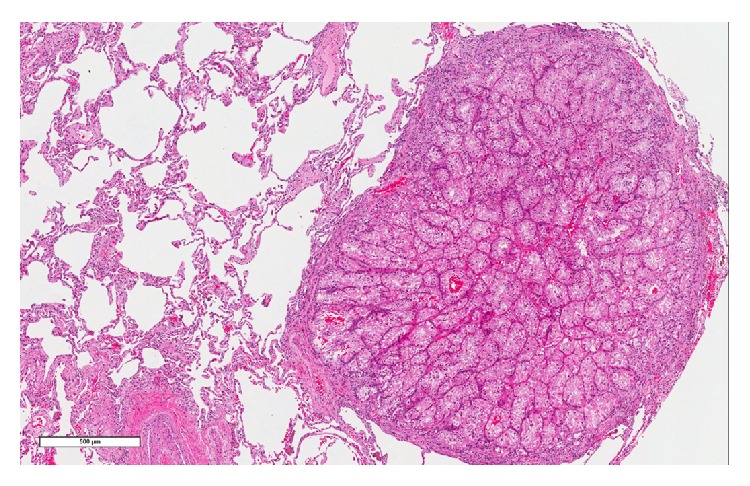
Metastatic renal cell carcinoma. Photomicrograph shows a metastatic focus (hematoxylin-eosin, original magnification ×4.7).

**Figure 3:**
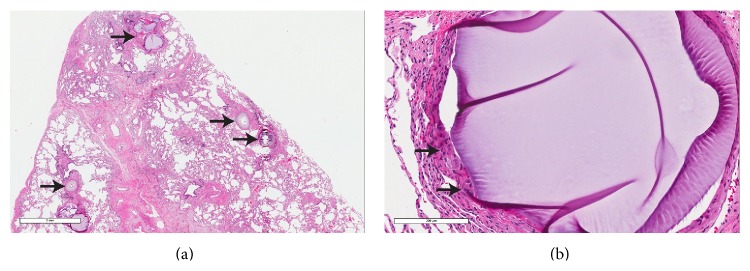
Trisacryl gelatin microemboli. (a) Microemboli (arrows) involving muscular pulmonary arteries (hematoxylin-eosin, original magnification ×1.4). (b) Microembolus with giant cells (arrows) on the left lateral aspect (hematoxylin-eosin, original magnification ×17.6).

**Figure 4:**
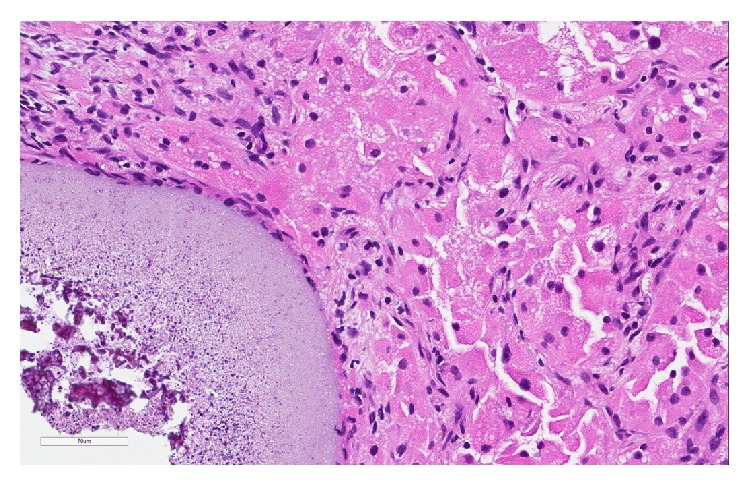
Colocalization of metastatic renal cell carcinoma and a trisacryl gelatin microembolus. The embolus is shown in the left lower corner (hematoxylin-eosin, original magnification ×20).

The patient underwent wedge resections of the lingula and left lower lobe and biopsy of a hilar lymph node. Histological sections of the lung showed multiple foci of metastatic RCC, measuring up to 1.0 cm in greatest dimension (Figure 2). In addition, numerous muscular pulmonary arteries contained trisacryl gelatin microemboli (Figure 3) and had medial hypertrophy consistent with pulmonary hypertension. Colocalization of metastatic RCC and trisacryl gelatin microspheres was noted (Figure 4). Sections of the hilar lymph node showed metastatic RCC.

## 3. Discussion

RAE is a minimally invasive procedure that can be utilized preoperatively for large, highly vascularized tumors to reduce the need for blood transfusion during surgery [2]. RAE is also an option for treatment of angiomyolipoma, congenital arteriovenous malformation, renal artery aneurysm, and pseudoaneurysm [3, 4]. Various embolic agents have been used, including metallic coils, polyvinyl alcohol particles, polyvinyl alcohol microspheres, ethanol, and absorbable gelatin sponge (Gelfoam; Pharmacia and Upjohn Co.). More recently, acrylic microspheres (Embosphere; BioSphere Medical, Inc.) have emerged as the new agent of choice [3].

RAE is considered safe and effective [3, 4]. A relatively common, but minor, complication is postinfarction syndrome, which develops within 72 hours and is characterized by pain, fever, and nausea [3, 4]. RAE may also be complicated by coil migration (when a metallic coil is used), incomplete embolization, and groin hematoma [3, 4]. Pulmonary complications have been described after transcatheter arterial embolization for hepatocellular carcinoma [5–7]. However, to our knowledge, pulmonary embolism following RAE with acrylic microspheres has not been reported previously.

The histological appearance of various embolic agents is known from gynecologic surgical specimens [8]. Pathologists should be aware of these findings and recognize them in both the targeted and nontargeted organs. Trisacryl gelatin microspheres are round and eosinophilic and have a folded appearance similar to thyroidal colloid. Polyvinyl alcohol particles are blue-gray and fibrillar. Contour SE, type of polyvinyl alcohol microspheres (Contour SE; Boston Scientific Corp.), are also blue-gray but have an oval contour; they show homogeneous outer halves with degenerate-appearing central areas. The tinctorial properties of these embolic agents are also different. Trisacryl gelatin microspheres are negative for periodic acid Schiff reaction and stain orange-pink with Movat pentachrome stain, whereas polyvinyl alcohol particles and microspheres are positive for periodic acid Schiff reaction and stain black with Movat stain. The endarterial foreign material in our case met the histological criteria for trisacryl gelatin microspheres.

It is possible that seeding of tumor cells occurred before the preoperative RAE procedure. Although there is no direct evidence, it is also possible that tumor cells, similar to trisacryl gelatin microspheres, entered the circulation during the RAE procedure or the subsequent nephrectomy. To prevent pulmonary embolism, Hirota et al. [9, 10] have advocated the placement of suprarenal inferior vena cava filters before RAE of unresectable RCC where there is tumor extension into the renal vein or inferior vena cava. Our case suggests that a wider indication for preventive measures should be cautiously considered.

## Abbreviations

RAE: Renal artery embolization
RCC: Renal cell carcinoma.
